# The Longitudinal Association of Lifestyle with Cognitive Health and Dementia Risk: Findings from the HELIAD Study

**DOI:** 10.3390/nu14142818

**Published:** 2022-07-08

**Authors:** Eirini Mamalaki, Sokratis Charisis, Costas A. Anastasiou, Eva Ntanasi, Kyriaki Georgiadi, Vassilis Balomenos, Mary H. Kosmidis, Efthimios Dardiotis, Georgios Hadjigeorgiou, Paraskevi Sakka, Nikolaos Scarmeas, Mary Yannakoulia

**Affiliations:** 1 Department of Nutrition and Dietetics, Harokopio University, 17671 Athens, Greece; emamal@hua.gr (E.M.); acostas@hua.gr (C.A.A.); 2 1st Department of Neurology, Aiginition Hospital, National and Kapodistrian University of Athens Medical School, 11528 Athens, Greece; scharissis@gmail.com (S.C.); e.ntanasi@hotmail.com (E.N.); ns257@cumc.columbia.edu (N.S.); 3 UT Health San Antonio, Department of Neurology, San Antonio, TX 78229, USA; 4 Department of Medical School, Democritus University of Thrace, 68100 Alexandroupolis, Greece; sandy.georgiadi@gmail.com (K.G.); balomenosv@gmail.com (V.B.); 5 Lab of Cognitive Neuroscience, School of Psychology, Aristotle University of Thessaloniki, 54124 Thessaloniki, Greece; kosmidis@psy.auth.gr; 6 School of Medicine, University of Thessaly, 41500 Larissa, Greece; edar@med.uth.gr; 7 Department of Neurology, Medical School, University of Cyprus, 2408 Nicosia, Cyprus; gmhadji@med.uth.gr; 8 Athens Association of Alzheimer’s Disease and Related Disorders, 11636 Marousi, Greece; vsakka@ath.forthnet.gr; 9 Taub Institute for Research in Alzheimer’s Disease and the Aging Brain, the Gertrude H. Sergievsky Center, Department of Neurology, Columbia University, New York, NY 10032, USA

**Keywords:** total lifestyle, cognition, dementia, Mediterranean diet, sleep, physical activity, functionality

## Abstract

The aim of the current study was to investigate whether a Total Lifestyle Index (TLI), including adherence to the Mediterranean diet, sleep duration, physical activity and engagement in activities of daily living, is associated with cognitive health over time and dementia risk, in a representative cohort of older people. A total of 1018 non-demented community-dwelling older adults ≥65 years old (60% women) from the HELIAD study were included. A comprehensive neurological and neuropsychological assessment was conducted at baseline and at the 3-year follow-up evaluating cognitive functioning, and a dementia diagnosis was set. Diet, physical activity, sleep duration and engagement in activities of daily living were assessed using standard, validated questionnaires at baseline. Sixty-one participants developed dementia at follow-up; participants who developed dementia were older and had fewer years of education compared with participants with normal cognition. With the exception of sleep duration, participants with normal cognition at follow-up scored higher in the individual lifestyle factors compared to those who developed dementia. Regarding TLI, values were lower for participants with dementia compared with those with normal cognition. Each additional unit of the TLI was associated with 0.5% of a standard deviation less decline per year of the Global Cognition score, whereas for each additional unit of the TLI, the risk for dementia was reduced by 0.2% per year (*p* < 0.05). Our results suggest that greater adherence to a healthy lifestyle pattern is associated with a slower decline of cognitive function and reduced dementia risk.

## 1. Introduction

Although dementia has emerged as a prevalent medical condition in the older age group [1], the evidence to date indicates that there is no effective cure and even the suggested therapies have limited efficacy [2]. Thus, data from observational studies and clinical trials focus on the role of primary prevention and the modifiable lifestyle factors that may delay the onset and the course of the disease [3]. Many studies have indicated a protective relationship between various aspects of lifestyle, such as diet, physical activity and sleep, with cognitive decline and the risk of dementia [4,5,6,7,8]. 

As the combination of lifestyle components on the risk of dementia may be quite strong overall [9], total lifestyle patterns have also been explored. For example, our research team, in a previous cross-sectional analysis, showed that a lifestyle index combining adherence to the Mediterranean diet, sleep quality, physical activity and engagement in activities of daily living, including social activities, was cross-sectionally associated with reduced odds for dementia, mild cognitive impairment and low global cognition [10]. Moreover, large-scale, prospective studies have shown that a favorable lifestyle, consisting of absence of smoking, regular physical activity, healthy diet and moderate alcohol consumption, has been associated with reduced dementia risk [11,12,13]. On the other hand, multi-domain interventions targeting aspects of lifestyle such as diet, physical activity, cognitive training and control of cardiovascular risk factors have yielded contradictory results [14,15,16,17].

Some of the studies on this topic lack a comprehensive neuropsychological assessment of the participants and have relied only upon rough assessments of cognitive function [9,11,12,13,18]. Moreover, they mainly focus on dementia and/or mild cognitive impairment risk [9,12], rather than the assessment of cognitive functioning and the differential rates of cognitive change over time. As dementia has a long preclinical period, the study of cognitive function is of great importance in the investigation of its pathophysiology. Finally, the majority of these indices have assumed monotonic effects between the components investigated and the cognitive function and have incorporated only a limited number of components, whereas important aspects of lifestyle, such as sleep and engagement in leisure time activities, have been, to a greater or a lesser extent, ignored. Thus, one may speculate that the controversial results of the clinical trials [14,15,16,17] could be attributed, to some extent, to the assumption of monotonic effects and the limited number of lifestyle factors incorporated in the indices. 

In an effort to address these limitations, we explored if an index including adherence to the Mediterranean diet, sleep duration, physical activity and engagement in activities of daily living may be associated with cognitive trajectories and dementia incidence in a representative sample of older adults. 

## 2. Materials and Methods

### 2.1. Sample and Procedures

The present sample was drawn from HELIAD (Hellenic Longitudinal Investigation of Aging and Diet), which is a population-based, multidisciplinary, collaborative study taking place in Greece and, specifically, in a suburb of Athens and an urban area, the city of Larissa (including its rural surroundings). The sample consisted of community-dwelling older people aged ≥65 years old, who were selected through random sampling from municipality registries. More details about the study design and data collection can be found in previously published works [19,20,21]. After the baseline evaluation, all participants are scheduled to be re-evaluated approximately every 3 years, performing some of the baseline assessments, whereas consensus diagnosis is conducted at every visit. To date, two evaluations per person have been completed (the baseline and the first follow-up). Detailed information regarding medical and family history, lifestyle and demographics was collected. Moreover, all participants received a thorough neurological and neuropsychological evaluation through structured questionnaires, psychometric tests and clinical evaluations. Adequately trained health professionals performed all assessments, apart from those involving self-assessment tools. In specific, neuropsychological evaluation was conducted by trained neuropsychologists, whereas neurological and clinical evaluation by certified neurologists. Furthermore, trained registered dieticians provided assistance to the study participants or their caregivers, when needed, for the completion of the dietary assessment tools. All health professionals provided assistance for the assessment of the other lifestyle factors, i.e., physical activity and engagement in activities of daily living. Assessments took place in day care centers for older people, the participants homes or municipal public health clinics. All participants gave written informed consent, and the study protocol was approved by the Ethics Committees of the University of Thessaly and the National and Kapodistrian University of Athens. 

### 2.2. Dietary Assessment

Dietary intake was assessed at baseline using a semi-quantitative Food Frequency Questionnaire, validated for the Greek population [22]. It consisted of 69 questions on the consumption of foods or combinations of foods, including dairy products, cereals, fruits, vegetables, meat, fish, legumes, added fats, alcoholic beverages, stimulants and sweets, during the previous month. Participants indicated the absolute frequency of consuming a certain amount of food using a 6-point scale (“never/rarely”, “1–3 times/month”, “1–2 times/week”, “3–6 times/week”, “1 time/day”, “≥2 times/day”). 

In order to evaluate adherence to the Mediterranean dietary pattern, we calculated an 11-item composite score from the answers to the Food Frequency Questionnaire, yielding the MedDietScore [23]. The scoring was based on the weekly consumption of 11 food groups (non-refined cereals, fruits, vegetables, legumes, potatoes, fish, meat and meat products, poultry, full-fat dairy, olive oil use and alcohol). Specifically, for the consumption of food groups assumed to represent healthful components of the Mediterranean diet, i.e., those with a recommended intake of 3 servings per week or more, such as non-refined cereals, fruits, vegetables, legumes, fish, potatoes and olive oil use, a score of 0 was assigned when the participants reported no consumption and scores 1–5 for rare to daily consumption. For the unhealthy food components of the pattern, scoring was assigned on a reverse scale, i.e., from 5 when someone reported no consumption to 0 for daily consumption. For alcohol intake, it was assumed that small amounts of consumption were characteristic of the Mediterranean diet, while either high or no consumption was assumed to diverge from this dietary pattern. Thus, a score of 5 was assigned for consumption of less than 300 mL of wine/day but more than 0 mL of wine/day, a score of 0 was assigned for no consumption or for consumption of more than 700 mL/day and scores of 1 to 4 were assigned for consumption of 600–700, 500–600, 400–500 and 300–400 mL/day, respectively. All alcoholic beverages were converted into ml of wine, assuming that 12 g of ethanol correspond to 100 mL of wine. The MedDietScore ranges between 0 and 55, with higher values indicating greater adherence to the Mediterranean diet. 

### 2.3. Physical Activity Assessment 

Physical activity assessment was conducted at baseline using the validated Athens Physical Activity Questionnaire (APAQ) [24]. Participants were asked about the time spent in occupational, household and recreational activities, sedentary and sleep during the last week. According to the specific metabolic equivalent (MET) corresponding to each of these activities, energy expenditure was calculated based on the participant’s body weight in kilograms divided by 60. The physical activity variable was expressed as total MET-min/day, excluding sleep.

### 2.4. Sleep Assessment 

Sleep habits were assessed at baseline through the Sleep Scale from the Medical Outcomes Study [25]. This is a self-report questionnaire consisting of 12 questions, with the timeframe being the previous month. For sleep duration, participants were asked to report how many hours they slept each night during the past 4 weeks; self-reported sleep duration was used as hours/night. 

### 2.5. Assessment of Engagement in Activities of Daily Living

Assessment of Engagement in Leisure Time Activities was performed at baseline using the IADL-extended scale, which evaluated functionality and capabilities relating to maintenance of self and lifestyle [26]. The scale contains 5 questions referring to cognitive leisure time activities (going to classes, participating in community volunteer work, going out to a club or cultural center activities, to the movies, a restaurant, a sporting event and visiting friends or relatives in the last month) and 4 questions referring to complex/advanced activities of daily living (having difficulties shopping, doing light housework, or getting around the neighborhood and needing help with medication). The possible answers to these questions were yes/no, with the total score ranging from 0 to 9, with higher values indicating greater engagement in the aforementioned activities. 

### 2.6. Neuropsychological Evaluation and Clinical Diagnoses

Neuropsychological evaluation was conducted, at both visits, by trained neuropsychologists with a comprehensive battery of neuropsychological tests, lasting approximately one hour, assessing cognitive functioning. Specifically, the tests used included the Mini-Mental State Examination [27], the Greek Verbal Learning Test (immediate and delayed recall) [28], the Medical College of Georgia Complex Figure Test (copy condition, immediate recall, delayed recall and recognition) [29], a semantic and phonological verbal fluency test [30], subtests of the Greek version of the Boston Diagnostic Aphasia Examination short form, namely, the Boston Naming Test short form, and selected items from the Complex Ideational Material Subtest [31], the Trail Making Test Part [32], an abbreviated form of Benton’s Judgment of Line Orientation [33], the Clock Drawing Test [34], anomalous sentence repetition [31], a graphical sequence task and motor programming [29].

In order to develop a composite score reflecting cognitive functioning, we took the raw scores on the individual neuropsychological tests from the participants who did not have dementia or mild cognitive impairment diagnosis and converted them into z-scores. Subsequently, we added these z-scores to compute a Global Cognition score.

Based on the neuropsychological assessment as well as the structured neurological examination, where neurological findings were documented, and after consensus meetings of all study investigators (neurologists and neuropsychologists), the diagnosis of dementia was determined where appropriate, according to international criteria [35,36].

### 2.7. Total Lifestyle Index Calculation

A Total Lifestyle Index (TLI) was computed using 4 components, namely adherence to the Mediterranean diet, sleep duration, physical activity and engagement in activities of daily living. Index calculation was based on a series of linear regression models exploring the association of the separate lifestyle factors (different index components) with baseline cognitive performance. In each model, the baseline cognitive score was the dependent variable, and the respective index component was the predictor. The models were adjusted for age, sex and years of education. In order to consider a potential non-linear association between the predictor (lifestyle factors) and the response variables (cognitive performance), the respective relationships were also modeled using restricted cubic splines with three knots placed at the 10th, 50th and 90th percentiles of the predictor variable [37]. The likelihood ratio test was then used to compare the model, including only the linear term with the model and including both the linear and cubic spline terms; if statistically significant, the second model was considered to be a better fit.

A value from 0 to 3 was assigned to each component based on the distribution of the model-predicted baseline cognitive performance. Specifically, for all components, a value of 0 was assigned for the range of values corresponding to the first quartile of the distribution of the baseline Global Cognition score (<25th percentile, lowest baseline cognitive function in relation to the specific component) and a value of 1, 2 or 3 was assigned for the range of values corresponding to the second (≥25th percentile and <50th percentile), third (≥50th percentile and <75th percentile) and forth (≥75th percentile, highest baseline cognitive function in relation to the specific component) quartiles of the distribution of the baseline Global Cognition score, respectively. Supplementary Figures S1–S4 present the distribution of the mean predicted Global Cognition score at baseline in relation to the separate lifestyle factors on which the construction of the TLI was based. Thus, as presented in the figures, the highest score of the TLI was assigned to those with moderate to high adherence to the Mediterranean diet, moderate sleep duration, moderate levels of physical activity and high engagement in activities of daily living. 

Thus, TLI, as the sum of all the sub-scores of the individual lifestyle factors examined, takes values from 0 to 12, with higher values indicating a cognitively beneficial lifestyle.

### 2.8. Outcomes

Cognitive health, assessed as Global Cognition Score, and dementia risk were the primary outcomes of the study and were evaluated as described above (neuropsychological evaluation and clinical diagnoses).

### 2.9. Statistical Analyses

Statistical analyses were performed using SPSS software and RStudio: Integrated Development Environment for R (RStudio, PBC). Type I probabilistic error was defined as *p* ≤ 0.05. Characteristics of the participants were expressed as mean values ± standard deviation or as percentages. Differences among groups were tested through analysis of variance for continuous variables and Pearson’s χ^2^ for categorical variables. For all analyses, participants diagnosed with dementia at baseline (*n* = 103) were excluded.

We constructed two series of Generalized Estimating Equations (GEE) models. The repeated measures for each participant were treated as a cluster; potential within-cluster correlations were considered by specifying an appropriate working correlation matrix (exchangeable—compound symmetry) based on the Quasi-likelihood under the independence model criterion. We used GEE models with a Gaussian probability distribution and an identity link function to evaluate the associations between TLI, as well as separate lifestyle factors and differential rates of cognitive change. Specifically, the Global Cognition score was the dependent variable, and the TLI, time (in years from baseline assessment) as well as the TLI × time interaction term were the predictors. TLI was used both as a continuous variable and as a discrete variable, based on the quintiles of its distribution. The 1st quintile, indicating the lowest lifestyle scores, served as the reference and was compared to the 2nd, 3rd, 4th and 5th quintiles, with the last one indicating the highest lifestyle scores. In subsequent similarly structured models, the predictors were the individual score subcomponents treated either as continuous variables or as index-derived quartiles, based on which TLI scores emerged, with the lowest values serving as the reference category. We also calculated GEE models with a binomial probability distribution and a logit link function. Dementia incidence was the dichotomous outcome, and the predictors were the same as in the GEE linear models. All the models included terms for the following possible confounders: age at baseline, sex and years of education. Age at baseline and years of education were treated as continuous variables. The results presented represent the estimates (beta coefficients or exponentiated beta coefficients for linear and logistic regression GEE models, respectively) of the interaction term TLI × time.

In an attempt to interpret in a clinically meaningful way the association of TLI with differential rates of cognitive change, we tried to estimate how one year increase in participants’ age influences the Global Cognition score and to compare it with the respective one from the TLI. Thus, we calculated an adjusted (for sex and years of education) GEE model with Global Cognition score as the dependent variable in the entire HELIAD study sample; participants completed the baseline and the follow-up evaluations (*n* = 1853) (data not shown). 

Finally, in the supplementary analyses, we also excluded participants diagnosed with dementia and mild cognitive impairment both at baseline and at follow-up, and we repeated the analyses for the associations of TLI, as a continuous variable and as quintiles, with differential rates of cognitive functioning.

## 3. Results

A total of 1018 participants without dementia at baseline completed both evaluations (baseline and follow-up) and had full data on all lifestyle factors (adherence to the Mediterranean diet, sleep duration, physical activity and engagement in activities of daily living). The baseline characteristics of all participants by the diagnosis of the follow-up are presented in Table 1. The mean interval between the baseline and the follow-up visit was 3.0 ± 0.8 years. In total, 61 participants developed dementia. Participants who developed dementia were older and had fewer years of education compared with participants with normal cognition. With the exception of sleep duration, participants with normal cognition at follow-up scored higher in the individual lifestyle factors compared to those who developed dementia. TLI values were lower for participants with dementia compared with those having normal cognition (Table 1). 

**Figure 1:**
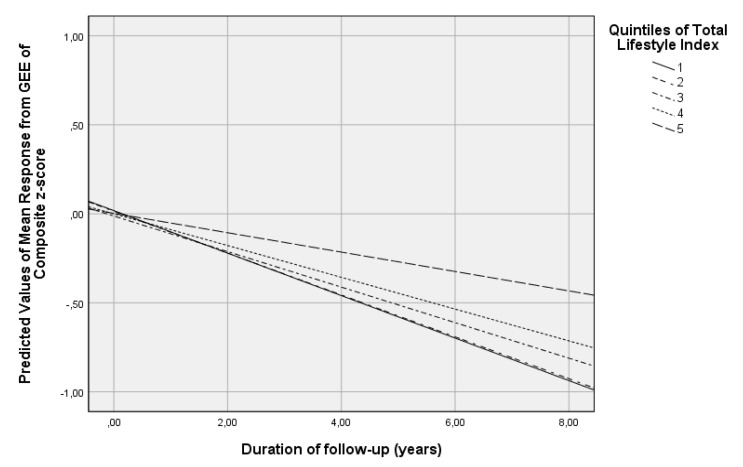
Results from Generalized Estimating Equations assessing the associations between the quintiles of the Total Lifestyle Index (independent variables) with differential rates of the Global Cognition score over time (dependent variables) in non-demented participants at baseline (*p* for trend < 0.001). All models were adjusted for age, sex and years of education.

Linear GEE models revealed that each additional unit of the TLI was associated with 0.5% of a standard deviation less decline per year in the Global Cognitive score (Table 2). The calculation of the relationship between aging and Global Cognition score indicated 6.1% of a standard deviation reduction of the composite score for each additional year of aging (β = −0.061, *p* < 0.001). This finding suggests that the biennial cognitive benefit of a 6-unit increase in the score of the TLI offsets the cognitive decline associated with one year increase in participants’ age. In comparison with participants with the lowest TLI values, those with the highest values had 5.8% of a standard deviation less decline per year on the Global Cognition score (Table 2, Figure 1).

TLI was associated with a significantly lower risk for dementia development. For each additional unit of the TLI, the risk for dementia was reduced by 0.2% per year of follow-up. In detail, participants in the highest quintile of the TLI (highest scores) had 3.7% less risk for developing dementia per year of follow-up compared with those in the lowest quintile, with a significant trend for a dose–response association (Table 3).

Regarding the associations among the quartiles of individual lifestyle factors, from the fitted models of the association between the baseline Global Cognition score with the individual lifestyle factors, the analyses revealed that those in the fourth index-derived quartile of adherence to the Mediterranean diet, sleep duration, physical activity and engagement in activities of daily living (meaning they had the highest baseline Global Cognition score in relation to the respective lifestyle factors) had 5.4%, 5.5%, 5.7% and 5.9% of a standard deviation less decline of the Global Cognition score per year (Table 4). 

Regarding the risk for developing dementia, each additional unit increase in the score indicating adherence to the Mediterranean diet, sleep duration, physical activity and engagement in activities of daily living was associated with 3.2%, 3.4%, 3.5% and 3.3% reduction in the risk for developing dementia per year of follow-up, respectively (Table 5).

In an even more conservative approach, participants with dementia and mild cognitive impairment at baseline and follow-up were also excluded, leaving only participants within the normal spectrum of cognitive function both at baseline and at follow-up. The results showed that each additional unit of the TLI was associated with 0.4% of a standard deviation less decline in the Global Cognition score per year of follow-up (Supplementary Table S1).

## 4. Discussion

The present longitudinal study showed that higher adherence to a lifestyle, combining adherence to the Mediterranean diet, sleep duration, physical activity and engagement in activities of daily living, was associated with a slower decline in cognitive function prospectively and reduced risk for dementia. These findings indicate that a lifestyle index, which is based on the baseline association of cognitive functioning with the individual factors, is longitudinally validated, as it is associated with cognitive health in the follow-up.

The construction of the index was based on both an *a priori* and an *a posteriori* basis; the *a-priori* basis lies in the fact that the individual lifestyle factors were selected by taking into account previous knowledge regarding the association of lifestyle factors with cognitive health, and the *a-posteriori* basis as it was constructed by taking into account the distribution of the Global Cognition score in relation with the individual lifestyle factors at baseline. Thus, one may argue that the index is population specific. On the other hand, this index was constructed based on relationships of lifestyle factors with cognitive performance in a random sample of community-dwelling individuals; assuming good sample representativeness, results might be generalizable to other similar populations. Although the optimal methodology for the construction of lifestyle indices, *a priori, a posteriori* or both, has yet to be identified [38], we believe that our index may have clinical implications and potential use in clinical practice as it is longitudinally evaluated. We showed that the biennial cognitive benefit of a 6-unit increase in the score of the TLI, which consisted of, to a greater or a lesser extent, modifiable lifestyle factors, offsets the cognitive decline associated with one year increase in age. The present findings are in accordance with those of previous studies that have shown that a combination of lifestyle factors is associated with reduced dementia risk [9,11,12]. However, the present study expands on previous studies by showing that except for dementia risk, lifestyle is also associated with a slower rate of decline in cognitive functioning. This suggests that lifestyle may offer potential benefits long before cognitive decline progresses into dementia. This is supported by the fact that the association between lifestyle and total cognitive function remained significant in the sub-sample of the participants within the normal spectrum of cognition (i.e., excluding participants with dementia and mild cognitive impairment both at baseline and at follow-up). 

Although some components of lifestyle, such as diet and physical activity, have been investigated in previously described indices, parameters, such as sleep and engagement in activities of daily living, including social activities, have not been explored in previous studies. We showed that a lifestyle index, which included components other than the basic lifestyle factors, was positively associated with cognitive health prospectively. The omission of these components may explain the partial failure of previous clinical trials [14,15,16,17]. In any case, the results found from the present study should be replicated in future clinical trials.

All of the aspects incorporated in the index, to a greater or a lesser extent, have been individually examined in relation to cognitive health. Specifically, adherence to the Mediterranean diet has been associated with reduced risk for developing dementia and with enhanced cognitive function [39,40]. Similarly, higher levels of physical activity can improve cognitive health, even in people living with dementia [41,42]. Short or long sleep duration has been related to a faster decline in cognitive functioning [43]. Finally, significant difficulties in the performance of activities of daily living have been reported, even in the very early stages of cognitive impairment [44]. Various mechanisms have been proposed for these associations, such as anti-oxidant and anti-inflammatory pathways and the promotion of cardiovascular health [45,46,47,48]. As the pathophysiology of dementia and cognitive decline is complex, the combination of lifestyle factors may have the potential to affect the aforementioned mechanisms at different levels, resulting in a stronger cumulative effect. Indeed, a recent clinical study showed that the combination of lifestyle factors had a reverse epigenetic impact on aging [49].

Finally, it should be noted that participants with the highest scores in engagement in activities of daily living had a 5.9% decreased risk of decline in cognitive functioning per year, whereas individuals with the highest TLI scores had 5.8% of a standard deviation less decline per year in the Global Cognitive score. This underlines the significance of engagement in activities of daily living in predicting cognitive function. In detail, as the questionnaire used included both engagement in leisure time activities (visiting friends or going to classes), as well as complex/advanced activities (having difficulties shopping or getting around the neighborhood), we believe that the association with cognitive functioning may be attributable to a large extent to the complex activities of daily living rather than the leisure time ones; older people with difficulties in complex activities of daily living may be in the pre-clinical stage of dementia [50]. On the other hand, when it comes to the prediction of dementia, total lifestyle appears to be a stronger predictor compared with individual components of the lifestyle, including engagement in activities of daily living.

In any case, it is important for health professionals to adopt a more holistic approach in assessing the lifestyle of older people; many aspects of lifestyle should be targeted as well as synergies between them, and not just some of the components, such as diet and/or physical activity. Similarly, policy makers need to gradually move from a single factor approach to total lifestyle recommendations for healthy living recommendations, as these could be more effective in terms of both outcomes and resources.

The present results should be interpreted bearing in mind the strengths and limitations of this research. Regarding limitations, the assessment of lifestyle factors was based on self-reported tools, which were prone to measurement error. However, objective methods are hard to use in a large-scale study. Additionally, all lifestyle factors were assessed at baseline, and changes over time were not considered in this study. Due to the relatively short follow-up interval, the issue of reverse causality cannot be entirely excluded. However, the fact that the observed associations persisted even after the exclusion of participants with baseline and follow-up dementia and mild cognitive impairment increased our confidence that reverse causality may not account for our findings. Moreover, we adjusted our models for important confounders, but the effect of other factors not assessed in this study, i.e., residual confounding, cannot be entirely excluded. Finally, although we detected significant associations between TLI and lifestyle factors with dementia, more dementia cases would allow stronger associations to emerge and be detected. 

In contrast, the study has several strengths, foremost of which is its longitudinal design, making it possible to gain potential insight into causality. All participants underwent a comprehensive neurological and neuropsychological evaluation, allowing us to study not only dementia, but also cognitive functioning. Another important strength is that we longitudinally validated an index that was constructed based on the highest predicted cognitive function at baseline, and we also took into consideration that the relationships between the different lifestyle factors and cognitive performance may diverge from linearity. Finally, the detailed assessment of various lifestyle components allowed us to account for lifestyle components not extensively examined in other studies with a similar aim.

## 5. Conclusions

In conclusion, in the present research, a healthy lifestyle, that included the adoption of a healthy plant-based diet, adequate sleep, engagement in physical activities and activities of daily living, was associated with slower cognitive decline and reduced dementia risk, even in participants within the completely normal spectrum of cognition. Health professionals should routinely assess lifestyle as a whole pattern, with intercorrelations and synergies between components, and target relevant changes for the prevention of cognitive impairment. 

## Figures and Tables

**Table 1:** Baseline demographic and lifestyle characteristics of the participants and by diagnosis at follow-up.

	All Participants *n* = 1018	Participants with Normal Cognition at Follow Up *n* = 957	Participants with Dementia at Follow-Up *n* = 61	*p*-Value *
Age (years)	73.1 ± 5.0	72.7 ± 4.9	77.4 ± 4.7	**<0.001**
Sex (% women)	59.7	59.9	57.1	0.692
Education (years)	8.2 ± 4.9	8.3 ± 4.9	6.0 ± 4.8 *	**<0.001**
Duration of follow-up (years)	3.0 ± 0.8	3.0 ± 0.9	3.0 ± 0.8	0.975
MedDietScore (0–55)	33.6 ± 4.5	33.7 ± 4.6	32.1 ± 3.5	**0.011**
Physical Activity (/ 200 MET-min/day)	7.5 ± 1.4	7.6 ± 1.4	7.0 ± 1.2	**0.002**
Sleep Duration (hours)	6.5 ± 1.4	6.5 ± 1.4	6.7 ± 2.0	0.501
Engagement in activities of daily living (0–9)	4.6 ± 1.3	4.7 ± 1.2	3.8 ± 1.4	**<0.001**
Total Lifestyle Index (0–12)	6.0 ± 4.4	6.2 ± 4.4	2.9 ± 3.8	**<0.001**

* refers to the comparison between participants with dementia and those with normal cognition (analysis of variance for continuous variables and Pearson’s χ^2^ for categorical variables). Continuous variables are presented as mean values ± standard deviation and categorical variables as relative (%) frequencies. MedDietScore: Score indicating adherence to the Mediterranean diet. Bold numbers indicate statistical significance (*p* < 0.005).

**Table 2:** Results from Generalized Estimating Equations assessing the association between Total Lifestyle Index as a continuous variable and as quintiles (independent variables) with differential rates of the Global Cognition score over time (dependent variables) in non-demented participants at baseline.

	Total Lifestyle Index as a Continuous Variable		Total Lifestyle Index as Quintiles			
	β (95% CI)	*p*	Quartiles	β (95%CI)	*p*	*p* for Trend
Global Cognition score	0.005 (0.003–0.007)	**<0.001**	1st (ref)			**<0.001**
			2nd	−0.002 (−0.0034–0.029)	0.881	
			3rd	−0.002 (−0.0040–0.036)	0.917	
			4th	0.017 (−0.015–0.048)	0.301	
			5th	0.058 (0.032–0.084)	**<0.001**	

All models were adjusted for age, sex and years of education. Bold numbers indicate statistical significance (*p* < 0.05), CI: Confidence Interval.

**Table 3:** Results from Generalized Estimating Equations that evaluated the association between Total Lifestyle Index as a continuous variable and as quintiles (independent variables) with dementia incidence (dependent variable) in non-demented participants at baseline.

	Total Lifestyle Index as a Continuous Variable		Total Lifestyle Index as Quintiles			
	RR (95% CI)	*p*	Quartiles	RR (95% CI)	*p*	*p* for Trend
Dementia incidence	0.998 (0.997–0.999)	**<0.001**	1st (ref)			**<0.001**
			2nd	0.974 (0.956–0.992)	**0.004**	
			3rd	0.965 (0.948–0.982)	**<0.001**	
			4th	0.981 (0.962–1.000)	**0.049**	
			5th	0.963 (0.949–0.978)	**<0.001**	

All models were adjusted for age, sex and years of education. Bold numbers indicate statistical significance (*p* < 0.05). RR: Relative Risk, CI: Confidence Interval.

**Table 4:** Results from Generalized Estimating Equations assessing the associations between quartiles of individual lifestyle factors based on the distribution of the Global Cognition score at baseline for each specific lifestyle factor (independent variables) with differential rates of Global Cognition score over time (dependent variable) in non-demented participants at baseline.

	Adherence to the Mediterranean Diet				Sleep Duration				Physical Activity				Engagement in Activities of Daily Living			
	Quartiles	β (95% CI)	*p*	*p* for Trend	Quartiles	β (95% CI)	*p*	*p* for Trend	Quartiles	β (95% CI)	*p*	*p* for Trend	Quartiles	β (95% CI)	*p*	*p* for Trend
Global Cognition score	1st (ref)			**<0.001**	1st (ref)			**<0.001**	1st (ref)			**<0.001**	1st (ref)			**<0.001**
	2nd	−0.010 (−0.040–0.021)	0.534		2nd	−0.005 (−0.035–0.025)	0.739		2nd	−0.002 (−0.032–0.028)	0.917		2nd	0.009 (−0.021–0.039)	0.576	
	3rd	0.018 (−0.010–0.047)	0.208		3rd	0.015 (−0.014–0.044)	0.313		3rd	0.012 (−0.017–0.042)	0.409		3rd	0.018 (−0.012–0.047)	0.238	
	4th	0.054 (0.030–0.078)	**<0.001**		4th	0.055 (0.031–0.079)	**<0.001**		4th	0.057 (0.032–0.081)	**<0.001**		4th	0.059 (0.035–0.084)	**<0.001**	

All models were adjusted for age, sex and years of education. Bold numbers indicate statistical significance (*p* < 0.05). CI: Confidence Interval.

**Table 5:** Results from Generalized Estimating Equations that evaluated the association between quartiles of individual lifestyle factors based on the distribution of composite z-score at baseline for each specific lifestyle factor (independent variables) with dementia incidence (dependent variable) in non-demented participants at baseline.

	Adherence to the Mediterranean Diet				Sleep Duration				Physical Activity				Engagement in Activities of Daily Living			
	Quartiles	RR (95%CI)	*p*	*p* for Trend	Quartiles	RR (95% CI)	*p*	*p* for Trend	Quartiles	RR (95% CI)	*p*	*p* for Trend	Quartiles	RR (95% CI)	*p*	*p* for Trend
Dementia incidence	1st (ref)			**<0.001**	1st (ref)			**<0.001**	1st (ref)			**<0.001**	1st (ref)			**<0.001**
	2nd	0.977 (0.961–0.994)	**0.007**		2nd	0.975 (0.958–0.992)	**0.004**		2nd	0.974 (0.957–0.991)	**0.003**		2nd	0.977 (0.960–0.994)	0.007	
	3rd	0.984 (0.967–1.001)	0.065		3rd	0.980 (0.963–0.997)	**0.025**		3rd	0.979 (0.962–0.997)	**0.021**		3rd	0.981 (0.964–0.998)	0.031	
	4th	0.968 (0.955–0.982)	**<0.001**		4th	0.966 (0.952–0.980)	**<0.001**		4th	0.965 (0.952–0.980)	**<0.001**		4th	**0.967 (0.954–0.981)**	**<0.001**	

All models were adjusted for age, sex and years of education. Bold numbers indicate statistical significance (*p* < 0.05). RR: Relative Risk. CI: Confidence Interval.

## Data Availability

Data available upon request.

## Supplementary Materials

The following supporting information can be downloaded at: https://www.mdpi.com/article/10.3390/nu14142818/s1, Figure S1: Best model fit exploring the association of adherence to the Mediterranean diet with baseline Global Cognition score; Figure S2: Best model fit exploring the association of sleep duration with baseline Global Cognition score; Figure S3: Best model fit exploring the association of physical activity with baseline Global Cognition score; Figure S4: Best model fit exploring the association of engagement in activities of daily living with baseline Global Cognition score; Table S1: Results from Generalized Estimating Equations assessing the association between Total Lifestyle Index as a continuous variable and as quintiles (independent variables) with differential rates of Global Cognition score over time (dependent variables), in non-demented, non- mild cognitive impairment participants at baseline and at follow-up.
