# Supplementary material for: The Longitudinal Association of Lifestyle with Cognitive Health and Dementia Risk: Findings from the HELIAD Study
Source: Nutrients. 2022 Jul 8;14(14):2818. doi: 10.3390/nu14142818 (PMC9320599; doi:10.3390/nu14142818)
Supplement: Supplementary file 1 [file nutrients-14-02818-s001.zip › nutrients-1787631-supplementary.pdf]

Supplementary Materials

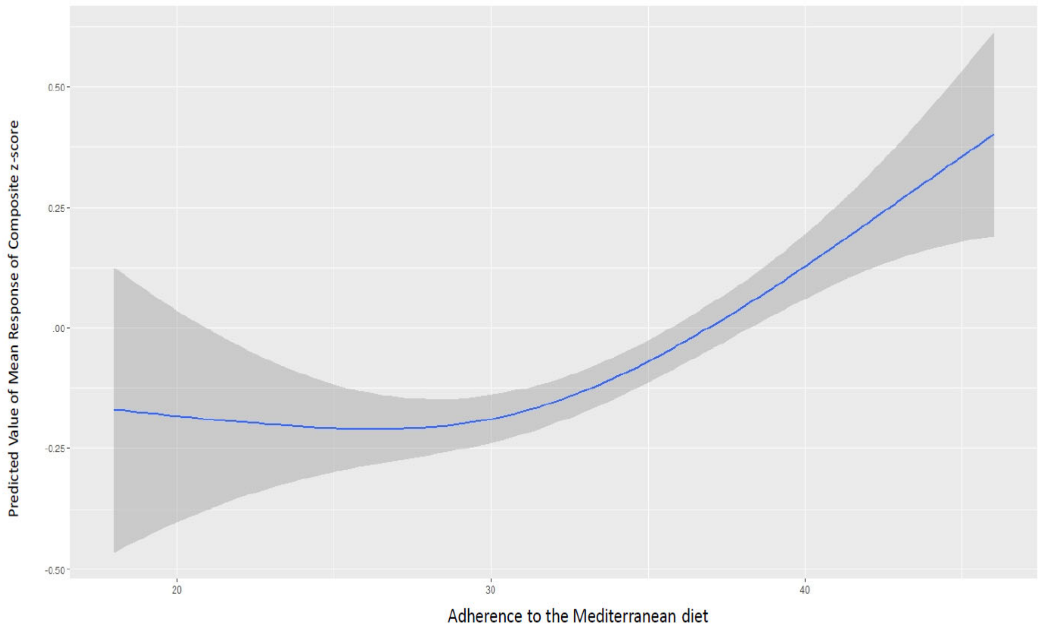

Figure S1. Best model fit exploring the association of adherence to the Mediterranean diet with baseline Global Cognition score. The model was adjusted for age, sex and years of education.

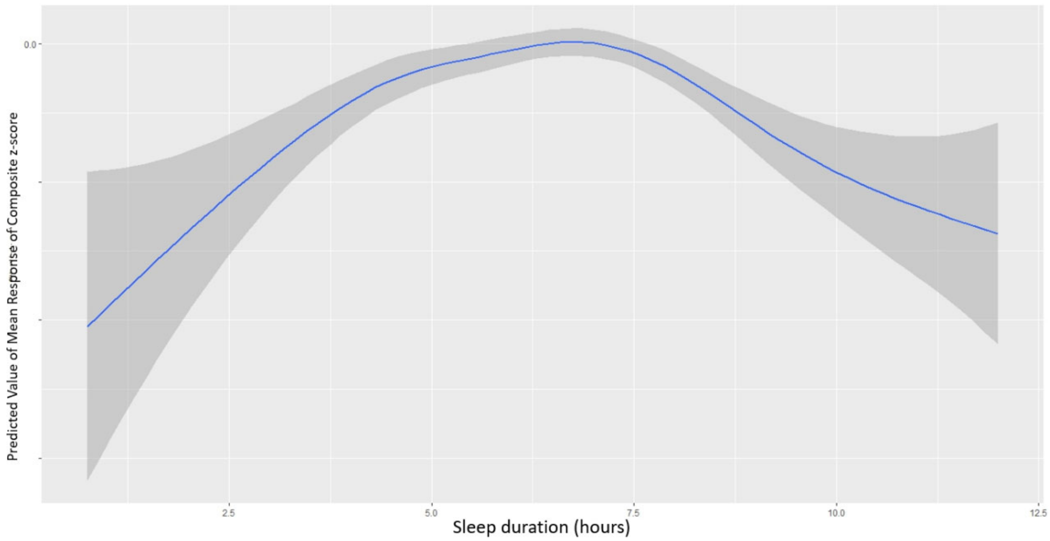

Figure S2. Best model fit exploring the association of sleep duration with baseline Global Cognition score. The model was adjusted for age, sex and years of education.

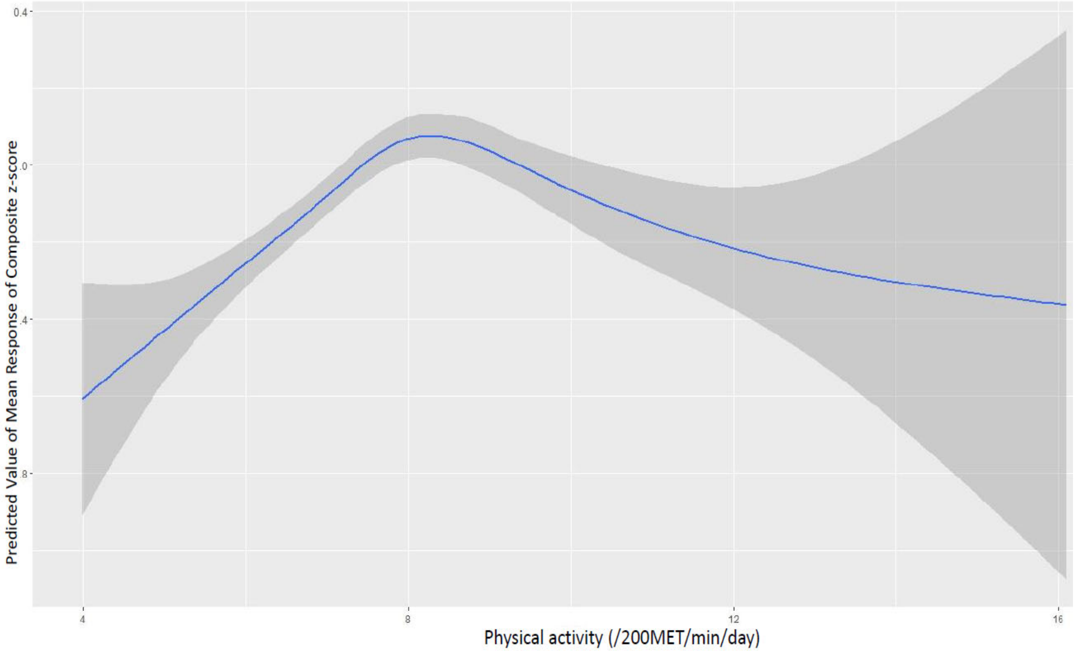

Figure S3. Best model fit exploring the association of physical activity with baseline Global Cognition score. The model was adjusted for age, sex and years of education.

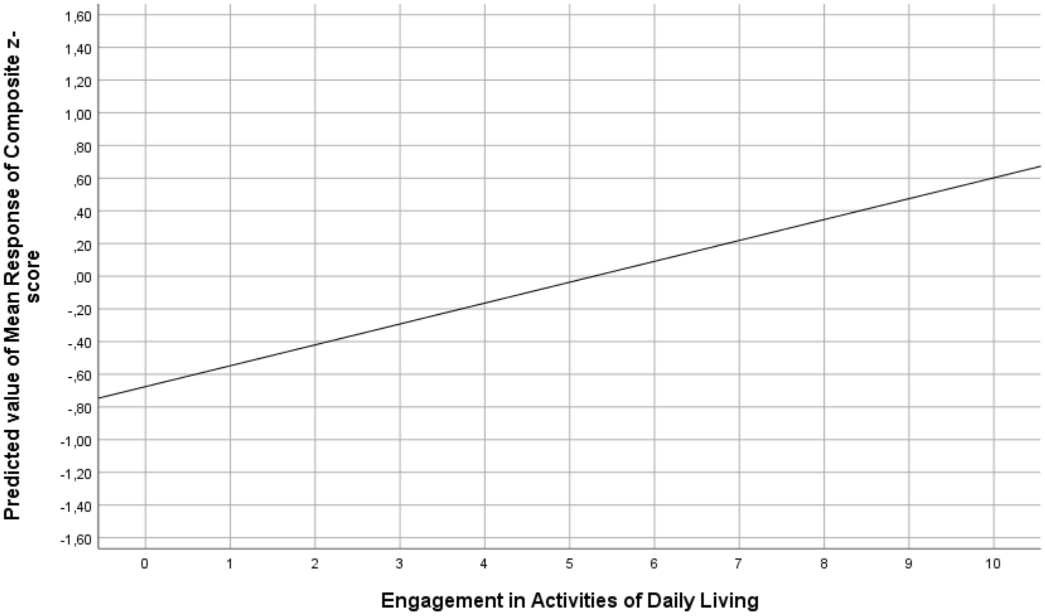

Figure S4. Best model fit exploring the association of engagement in activities of daily living with baseline Global Cognition score. The model was adjusted for age, sex and years of education.

Table S1. Results from Generalized Estimating Equations assessing the association between Total Lifestyle Index as a continuous variable and as quintiles (independent variables) with differential rates of Global Cognition score over time (dependent variables), in non-demented, non- mild cognitive impairment participants at baseline and at follow-up.

	**Total Lifestyle Index as a continuous variable**		**Total Lifestyle Index as quintiles**			
	**β (95% CI)**	***p***	**Quartiles**	**β (95% CI)**	***p***	***p* for trend**
Global Cognition score	0.004 (0.002-0.007)	**<0.001**	1st (ref)			**<0.001**
			2nd	-0.013 (-0.053-0.028)	0.545	
			3rd	-0.020 (-0.097-0.026)	0.391	
			4th	0.019 (-0.020- 0.058)	0.343	
			5th	**0.041 (0.007-0.075)**	**0.018**	

All models were adjusted for age, sex and years of education. Bold numbers indicate statistical significance ($p < 0.05$). CI: Confidence Interval.
